# Immunomodulatory Effect and an Intervention of TNF Signalling Leading to Apoptotic and Cell Cycle Arrest on ORL-204 Oral Cancer Cells by Tiger Milk Mushroom, *Lignosus rhinocerus*

**DOI:** 10.17113/ftb.60.01.22.7296

**Published:** 2022-03

**Authors:** Hui Yeng Yeannie Yap, Boon Hong Kong, Chee Sum Alvin Yap, Kien Chai Ong, Rosnah Binti Zain, Soon Hao Tan, Zuraiza Mohamad Zaini, Szu Ting Ng, Chon Seng Tan, Shin Yee Fung

**Affiliations:** 1 Department of Oral Biology and Biomedical Sciences, Faculty of Dentistry, MAHSA University, Jalan SP 2, Bandar Saujana Putra, 42610 Jenjarom, Selangor, Malaysia; 2 Medicinal Mushroom Research Group (MMRG), Department of Molecular Medicine, Faculty of Medicine, Universiti Malaya, 50603 Kuala Lumpur, Malaysia; 3 Department of Biomedical Sciences, Faculty of Medicine, Universiti Malaya, 50603 Kuala Lumpur, Malaysia; 4 Department of Oral Pathology and Oral Medicine, Faculty of Dentistry, MAHSA University, Jalan SP 2, Bandar Saujana Putra, 42610 Jenjarom, Selangor, Malaysia; 5 Oral Cancer Research and Coordinator (OCRCC), Faculty of Dentistry, Universiti Malaya, 50603 Kuala Lumpur, Malaysia; 6 Department of Oral and Maxillofacial Clinical Sciences, Faculty of Dentistry, Universiti Malaya, 50603 Kuala Lumpur, Malaysia; 7 LiGNO Biotech Sdn. Bhd., Jalan Perindustrian Balakong Jaya 2/2, Taman Perindustrian Balakong Jaya 2, 43300 Balakong Jaya, Selangor, Malaysia

**Keywords:** *Lignosus rhinocerus*, oral cancer, apoptosis, cell cycle, COX-2, MIP2

## Abstract

**Research background:**

Tiger milk mushroom (*Lignosus rhinocerus*) is a medicinal mushroom that is geographically distributed in the region of South China, Thailand, Malaysia, Indonesia, Philippines and Papua New Guinea. Consumption of its sclerotium has been reported to treat various ailments. However, its anticancer potential towards oral cancer cell lines is yet to be determined considering the traditional method of its consumption by biting/chewing of the sclerotium.

**Experimental approach:**

Mushroom sclerotial powder of cultivar TM02® was extracted and fractionated in a chromatographic column prior to cytotoxicity testing against a panel of human oral cancer cell lines. The capability of the identified bioactive fraction in regulating several molecules associated with its tumour necrosis factor (TNF) pathway was investigated.

**Results and conclusions:**

2,5-Diphenyl-2H-tetrazolium bromide (MTT) proliferation assay indicated that cell lines ORL-48 (derived from gingiva), ORL-188 (derived from the tongue) and ORL-204 (derived from buccal mucosa) were inhibited by cold water extract of *L. rhinocerus* sclerotia and its high-molecular-mass fraction (HMM) in varying degrees with ORL-204 being most affected. Hence, the treatment of ORL-204 with HMM mushroom extract was further investigated. HMM mushroom extract induced apoptosis and G_0_/G_1_ phase cell cycle arrest through caspase-3/7 cleavage. Activities of MIP2 and COX-2 were downregulated by 0.2- and 4.6-fold respectively in the HMM mushroom extract-treated ORL-204 cells.

**Novelty and scientific contribution:**

Using ORL-204, we showed that HMM mushroom extract may act *via* the TNF pathway at various network sites as a potential dietary compound for cancer prevention and natural adjunct therapeutic to conventional cancer treatment.

## INTRODUCTION

*Lignosus rhinocerus* or tiger milk mushroom (also known locally as ‘cendawan susu harimau’) has well-recorded medicinal values (*1*). Its sclerotium is traditionally used as a health tonic or treatment regime for asthma, bronchitis, various cancer ailments as well as discomforts caused by fright, fever, cough, vomiting or injury (*2*, *3*). It is consumed in the form of decoction, in a betel quid, and other preparations where the sclerotium is pounded with raw rice, infused and then taken as a drink (*4*, *5*). A technique mimicking cold water extraction has been described by Chan (*6*) where the sclerotium is grated on a hard surface such as granite plate with some water and the resulting mixture is further diluted with water before consumption. There is also a practice of biting/chewing of the sclerotium by local indigenous communities during their journeys in the wild (*7*).

Previous omics studies reported the presence of lectins, fungal immunomodulatory proteins, superoxide dismutase, aegerolysin and laccases in *L. rhinocerus* that could be involved in various bioactivities, including immunomodulatory properties (*8*-*10*), which play a pivotal role in diseases such as cancer. In fact, the anticancer properties of *L. rhinocerus* in numerous cell lines have previously been reported. A polysaccharide-protein complex from *L. rhinocerus* sclerotium has been shown to inhibit the growth of several leukaemic cell lines induced by a G_1_ phase cell cycle arrest (*11*), while a cold aqueous extract preparation derived from cultivar KUM61075 exhibited cytotoxicity against a panel of human cancer cell lines. The cytotoxic component(s) was/were speculated to be thermolabile, water-soluble protein/peptide(s) (*12*). On the other hand, Lee *et al.* (*13*) demonstrated the anticancer properties of a cold water extract of TM02® sclerotia, a protein- and carbohydrate-rich extract, against breast cancer MCF7 and lung cancer A549 cell lines. The efficacy of its molecular mass fractions and a partially purified cytotoxic serine protease-like protein against MCF-7 cells *via* a cross-talk in between intrinsic and extrinsic apoptotic routes has also been reported (*14*). However, despite the growing scientific data of beneficial therapeutic effects of *L. rhinocerus* against various cancer cell lines, its anticancer potential towards oral cancer cell lines remains unknown. In this study, we investigate the anticancer activity of TM02® sclerotial extracts on a panel of human oral cancer cell lines and their possible mode of action.

Oral cancer is selected as it is one of the more common cancers in the world with estimated  354 864 new cases diagnosed, and 177 384 deaths reported in the year 2018 (*15*). Furthermore, tiger milk mushroom has been consumed by chewing and kept in mouth for a considerable amount of time, prior to swallowing. Hence, it is intriguing to find out its cytotoxicity in the oral cavity. More than 90% of oral cancer that occur on the lips and in the oral cavity are squamous cell carcinoma (*16*). It is believed that oral squamous cell carcinoma (OSCC) develops through stages, from increasing severity of histological changes of premalignant lesions to malignancy. OSCC is life-threatening and with a mere five-year survival rate for stages 3 and 4. Early signs of oral cancer often go unnoticed and have been frequently discovered during routine dental examinations. Many cases of oral cancer may have advanced to an untreatable stage where the cancer cells have become aggressive and unresponsive to therapeutic drugs (*17*, *18*). In general practice, oral cancer is treated with either surgery, radiotherapy and/or chemotherapy. The treatment outcomes may include disease recurrence and post-treatment morbidity owing to the non-specific damages of these treatments to the function of healthy cells. Several determining factors that may increase the risk of cancer have been identified and these include massive exposure to chemical carcinogens such as tobacco and alcohol, solar ultraviolet radiation by excessive exposure of lips to the sun, human papillomavirus infection and a weakened immune system (*19*). Therefore, research that focused on natural immunomodulators to impede side effects of cytotoxic drugs has been gaining limelight in recent years. Natural compounds with simultaneous targeting of cancer pathways may often result in efficient and selective killing of cancer cells, which could be an added advantage for treatment of the disease (*20*, *21*). Thus, it is of tremendous interest if the anticancer properties reported for this medicinal mushroom are also effective for oral cancer.

## MATERIALS AND METHODS

### Extraction and fractionation of TM02® sclerotial powder

The freeze-dried sclerotial powder TM02® (reg no. MAL 11035004TC) was provided by LiGNO™ Biotech Sdn. Bhd. (Balakong Jaya, Selangor). Preclinical toxicological study determined that the product was not associated with any toxicity concerns. No-observed-adverse-effect level dose was more than 1000 mg/kg. The powder also did not cause detectable adverse effect on rats’ fertility, teratogenic and genotoxicity effects (*22*). Hot water, cold water and methanol extractions were carried out in a mass to volume ratio 1:20 (g/mL) as described earlier (*23*). Cold water extract of TM02® sclerotia was further fractionated by Sephadex® G-50 (fine) (Sigma-Aldrich, Merck, St. Louis, MO, USA) gel filtration chromatography column equilibrated with 0.05 M ammonium acetate (Sigma-Aldrich, Merck) buffer. Eluted fractions were subsequently grouped based on their molecular masses.

### Cell culture and maintenance

ORL-48, ORL-204 and ORL-188 oral cancer cell lines isolated respectively from gingiva (gum), buccal mucosa (lining of the cheeks and back of the lips) and tongue were obtained from Cancer Research Malaysia (Subang Jaya, Selangor). These cells were established from surgically resected specimens obtained from untreated primary human oral squamous cell carcinomata of the oral cavity as *in vitro* models to study a disease prevalent in Asia. Their growth characteristics, epithelial origin and molecular alterations were previously characterized (*24*, *25*). Genetic information and clinical data associated with these ORL cell lines are available at https://genipac.cancerresearch.my/ (*26*). ORL-48, ORL-204 and ORL-188 cells were cultured and maintained in DMEM/F-12 medium (Nacalai Tesque Inc., Kyoto, Japan) supplemented with 10% foetal bovine serum (Nacalai Tesque Inc.) and 0.1% penicillin-streptomycin (Sigma-Aldrich, Merck) at 37 °C and 5% CO_2_.

### MTT cytotoxicity assay

The 3-(4,5-dimethylthiazol-2-yl)-2,5-diphenyl-2H-tetrazolium bromide (MTT) assay was used to determine the antiproliferative activity of the extracts, where the yellow tetrazolium dye was reduced to purple crystalline formazan in metabolically active viable cells by NAD(P)H-dependent cellular oxidoreductases. ORL cell lines were seeded in monolayer and were allowed to adhere overnight prior to treatment with TM02® samples at different concentrations from 31.25 to 1000 µg/mL. Following 72 h of incubation, 20 μL of 5 mg/mL MTT solution (Calbiochem®, Sigma-Aldrich, Merck, San Diego, CA, USA) in phosphate buffered saline (Oxoid, Basingstoke, UK) were added to each well. The plate was then incubated for 4 h at 37 °C to promote the formation of purple formazan crystals. All the solutions were then aspirated and 200 μL of dimethyl sulfoxide (Sigma-Aldrich, Merck, St. Louis) were added to dissolve the attached formazan crystals. Absorbance was read at 570 nm after incubation for 10 to 30 min in the dark. Cell viability (%) was calculated and plotted against the extract concentration curve.

### Caspase activity measurement

Caspase-3/7, -8 and -9 activities were measured using respective Caspase-Glo® 3/7, Caspase-Glo® 8 and Caspase-Glo® 9 assay systems (Promega, Fitchburg, WI, USA) according to the manufacturer’s protocol. Cells were seeded in monolayer into a 96-well white plate and treated with high-molecular-mass (HMM) fractions of TM02® cold water extract at 75 µg/mL for 24, 48 and 72 h. After treatment, luminescent signal proportional to caspase activity was measured an hour after the addition of Caspase-Glo® reagent, which relies on the properties of a proprietary thermostable luciferase (Ultra-Glo™ Recombinant Luciferase, Promega), to the post-treated cells in 1:1 ratio.

### Flow cytometry analysis of cell cycle

Cell cycle distribution of treated cells was quantified using Muse™ cell cycle kit (Millipore, Burlington, MA, USA) according to the manufacturer’s protocol. In brief, cells were treated with HMM extracts at 40 and 250 µg/mL (IC_75_) for 72 h prior to 70% ethanol fixation and staining with Muse™ cell cycle reagent, a nuclear DNA stain containing propidium iodide, prior to the analysis on Muse™ cell analyzer (Millipore). The number of cells (in %) in the G_0_/G_1_, S and G_2_/M cell cycle phases, which differ in DNA content, were quantified by fluorescence-activated cell sorting (FACS) analysis with the configuration of 532 nm green laser line and three detection channels.

### ELISA assay

Selected modulators (TIMP1 and MIP2) were quantified using ELISA kit (Elabscience, Wuhan, PR China) according to the manufacturer’s protocol. In brief, cells were treated for 72 h with 10 (IC_25_) and 40 µg/mL of HMM extract. A total of 100 μL of standard or sample (the collected spent media from treated cells) was then added into each pre-coated well and incubated for 2.5 h at 37 °C to combine with the specific antibody. Specific biotinylated detection antibody was then added into each well and incubated for 1 h at 37 °C. This was followed by washing three times and then the addition of avidin-horseradish peroxidase conjugate for an hour incubation. Unbound components were washed off and substrate solution was added to each well. The enzyme-substrate reaction was terminated by the addition of stop solution (Elabscience). Absorbance was measured at 450 nm using Epoch microplate spectrophotometer (BioTek Instruments Inc., Winooski, VT, USA).

### Cyclooxygenase assay

Cyclooxygenase (COX) activity assay kit (fluorometric), purchased from Abcam, Cambridge, UK, served to detect the peroxidase activity of COX. A fresh set of standards was prepared and the supernatant of cells treated with 40 µg/mL of HMM extract for 72 h was collected according to the manufacturer’s protocol. Samples were kept on ice for downstream processing. Standard and reaction wells of samples and positive control were prepared and 10 µL of diluted arachidonic acid/NaOH solution were added into each reaction well. Fluorescence was measured (Ex/Em=535/587 nm) in a kinetic mode once every 15 s for 30 min.

### Statistical analysis

SPSS Statistics v. 21.0 (*27*) with one-way ANOVA followed by LSD’s *post hoc* test for multiple comparisons was used to compare the mean values. A p-value of less than 0.05 was considered as statistically significant.

## RESULTS AND DISCUSSION

In this study, pulverized *Lignosus rhinoceros* TM02® was extracted with hot water, cold water and methanol to coerce its carbohydrates, proteins and secondary metabolites, respectively. The yield and composition of these extracts were previously reported by our lab (*23*). Hot water extract consisted almost entirely of carbohydrates with 56% α-glucans, while cold water extract retained most of the extractable proteins as the extraction at low temperature of 4 °C had prevented the excessive degradation of thermolabile constituents including proteins and peptides. The major constituent of carbohydrates in the cold water extract was mainly glucose, which makes up the glucans in linear polysaccharides with 1,4-linkage. On the other hand, methanol extract consisted mainly of secondary metabolites (terpenoids) with no detectable level of proteins (*23*, *28*).

**Fig. 1:**
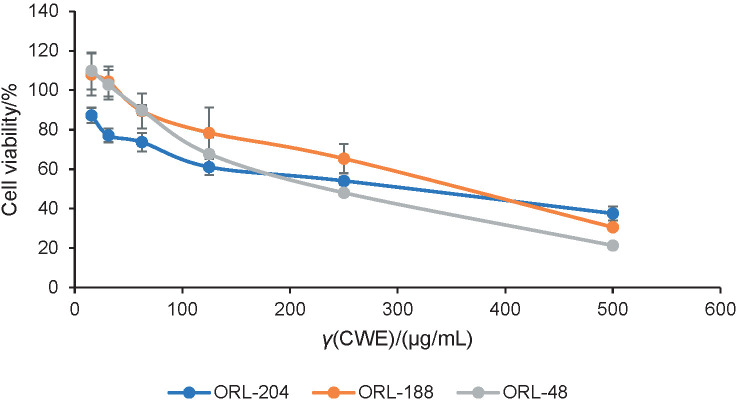
Cytotoxicity of *Lignosus rhinocerus* TM02® sclerotial cold water extracts after 72 h of treatment of ORL-48, -188 and -204 cells. Viability of the cells was determined by calculating the relative cell viability in MTT assay. Values are mean±S.D. CWE=cold water extract of TM02® sclerotia

The ORL-48, ORL-204 and ORL-188 oral squamous cell carcinoma (OSCC) cell lines were selected as they represent some of the most common areas for oral cancer with 100% orthotopic take rate and are highly hyperplastic (*24*). These cells were treated with 31.25−1000 µg/mL hot water, cold water and ethanol extracts for 72 h. Cytotoxic effect of the crude extracts was then measured with MTT assay. The reduction in the number of cells indicates inhibition of cell growth, and their sensitivity to drugs is further specified as the drug concentration needed to reach 50% inhibition of cell growth (IC_50_). ORL-48 and ORL-204 were more responsive towards cold water extract (Fig. 1) and the observed differences could be attributed to genetic predisposition resulting from site variation as ORL-48 and ORL-204 were isolated from gingiva and buccal mucosa while ORL-188 was extracted from tongue (*24*).

Owing to its antiproliferative potential, cold-water extract was further fractionated *via* Sephadex® G-50 column to high-, medium- and low-molecular mass (termed HMM (>30 kDa), MMM (7–30 kDa) and LMM (<7 kDa), respectively) extracts. In our previous study, we reported that HMM extract contained the highest amount of carbohydrates and proteins, without detectable level of β-glucans, suggesting the existence of polysaccharide-protein complexes in the fraction. Our group reported that HMM extract contained the glycosidic linkage (1,3,6-Glcp) in the prospective polysaccharide-protein complexes (*28*). On the other hand, LMM extract had a lower amount of carbohydrates with very few proteins, but higher number of secondary metabolites such as phenolics and terpenoids. MMM extract had a moderate amount of the macromolecules and secondary metabolites. It contained highly branched glucans with mixed linkages including 1,4,6-, 1,3,6- and 1,2,4,6-Glcp (*28*, *29*).

HMM, MMM and LMM extracts were tested on the OSCC cell lines with concentrations ranging from 16 to 500 µg/mL for 72 h (Table 1). A lower concentration range of crude extracts was selected since their stronger toxicity was expected. The range of IC_50_ values was determined to be 40−115 μg/mL for HMM extract, 125−175 μg/mL for MMM extract and above 400 μg/mL for LMM extract. HMM extract was found to be more cytotoxic to OSCC cell lines, specifically ORL-204, which originated from buccal mucosa cancer patient. Further testing of these extracts and fractions on an in-house isolated primary human fibroblast culture indicated that they are not toxic to normal tissues (Table 1). As HMM extract consists mainly of carbohydrates and proteins, the present result suggests that the bioactive component(s) responsible for its cytotoxic activity could be of proteoglycan nature and/or carbohydrate-protein complex derivatives.

**Table 1:** Cytotoxicity of *Lignosus rhinoceros* TM02® against various human cell lines

Mushroom extract	IC_50_			
ORL-48	ORL-188	ORL-204	Fibroblast
CWE	230	360	310	>500
HMM	115	135	40	>250
MMM	125	245	175	>250
LMM	>400	>400	>400	NP

IC_50_=half maximal inhibitory concentration value. It was determined from a mean plot of cell viability (in %) against concentration curve (*N*≥2). CWE=cold water extract, HMM=high molecular mass, MMM=medium molecular mass, LMM=low molecular mass, NP=not performed

**Fig. 2:**
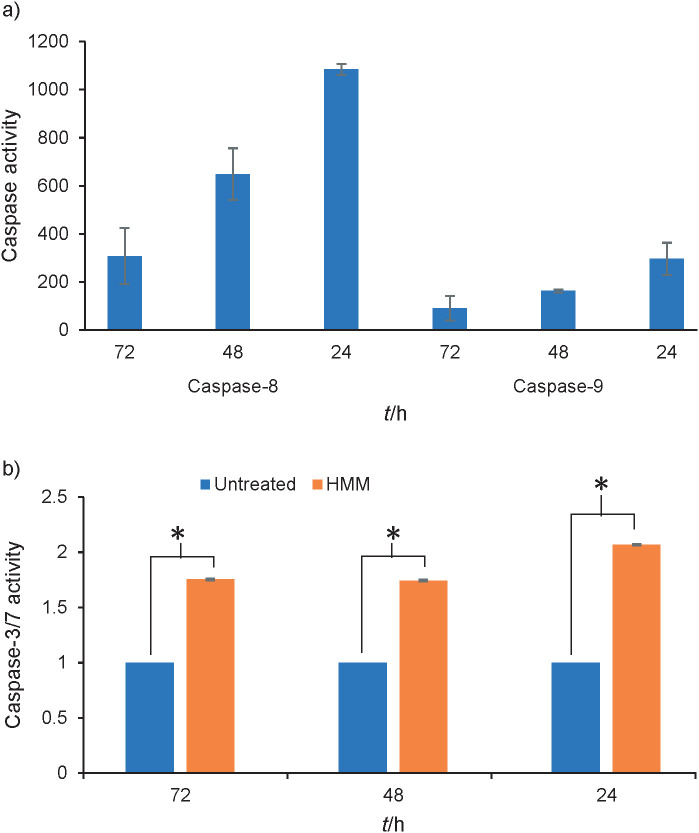
Upregulation of: a) caspase-8, -9 and b) -3/7 activities in ORL-204 cells after treatment with high molecular mass (HMM) fraction at 75 μg/mL over a period of 72 h. Data were expressed as fold change compared to the untreated control which was set as 1 (mean value±S.D., *N*=2; *p<0.05)

**Fig. 3:**
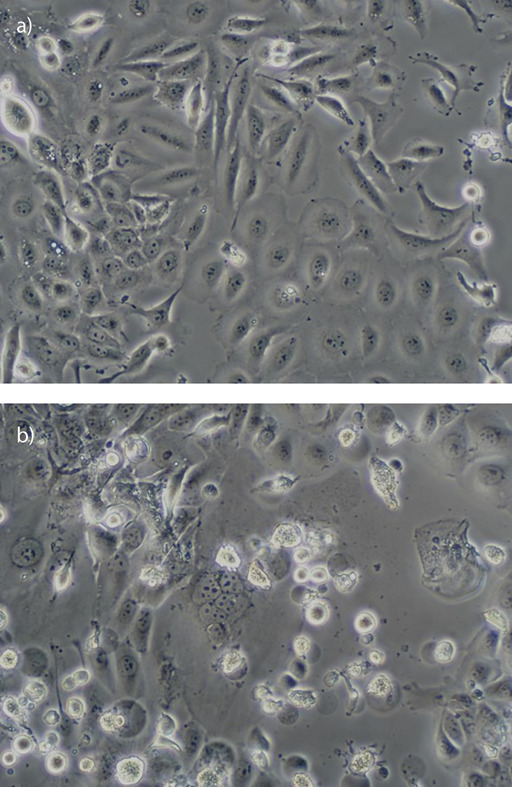
Cell morphology alterations of ORL-204 cells: a) control, and b) treated with 40 μg/mL of high molecular mass fraction for 72 h

We further determined if HMM extract induced apoptosis in ORL-204 by investigating the regulation of the key effectors in cell death signalling pathway. ORL-204 cell line was treated with HMM extract at 75 µg/mL (35 µg/L above IC_50_) for 24, 48 and 72 h. This concentration was selected for better capture of caspase activity that spans from 24 h onwards. At 24 h, HMM extract increased caspase-8 and -9 activities significantly in the treated cells (Fig. 2a). It is predicted that the active caspase-8 and -9 had subsequently activated the downstream executioner caspase as demonstrated in Fig. 2b, where caspase-3/7 activity increased up to 2-fold over a period of 72 h in HMM extract-treated cells as compared to the untreated control. This suggests that HMM extract induced apoptosis in a caspase-dependent manner *via* both the extrinsic and intrinsic signalling pathways. The cleavages of caspase-3/7 will presumably lead to the activation of endonuclease and protease as well as a series of cytomorphological changes including chromatin condensation and nuclear fragmentation (*30*). This is supported by higher numbers of apoptotic bodies (manifested as cell morphology alterations in the form of shrunken and fragmented cells) in ORL-204 treated with HMM extract at 40 µg/mL (IC_50_) for 72 h (Fig. 3).

Apoptosis is often linked to proliferation as they share the same set of regulators such as c‐Myc, p53, pRb, Ras, PKA, PKC, Bcl‐2, NF‐κB, CDK, cyclins and CKI (*31*). This knowledge prompted us to look into the cell cycle profile of ORL-204. Cells were treated with HMM extract at IC_50_ and IC_75_ (250 µg/mL) for 72 h prior to staining and data acquisition *via* FACS analysis. Cell death is prominent in the group of cells treated with HMM extract at IC_75_, with the accumulation of cells in the sub-G_0_/G_1_ peak, which may be indicative of DNA fragmentation due to apoptosis (Table 2). Furthermore, our earlier demonstration of upregulated caspase-3 activities in HMM extract-treated cells is indicative that the extract can mediate the cleavage and inactivation of p21 that converts cancer cells from growth arrest to apoptosis (*32*). HMM extract arrested ORL-204 at G_0_/G_1_ phase in the cell cycle where there was a minor but statistically significant increment of the cell population and subsequently a decreasing trend in the G_2_/M phase (Table 2). OSCC cell lines have been shown to overexpress cdk4 and cdk6, the key players in G_1_ phase (*33*), thus suggesting that these cell lines are more sensitive to G_1_ inhibitor. HMM extract may act as a cdk inhibitor that impedes downstream functions.

**Table 2:** Effect of high molecular mass extract on ORL-204 cell cycle distribution

Cell treatment	*N*(cells)/%		
G_0_/G_1_ phase	S phase	G_2_/M phase
Untreated	43.8±1.2	11.2±2.5	37.8±1.3
IC_50_	(47.5±2.3)*	12.4±3.0	(23.1±3.2)*
IC_75_	(49.1±1.7)*	13.6±2.9	(17.9±1.9)*

Cells were treated with 40 (IC_50_) and 250 µg/mL (IC_75_) of HMM extract for 72 h. Cell distribution (%) in G_0_/G_1_, S and G_2_/M phases is expressed as mean value±S.D. (*N*=3; *p<0.05)

**Fig. 4:**
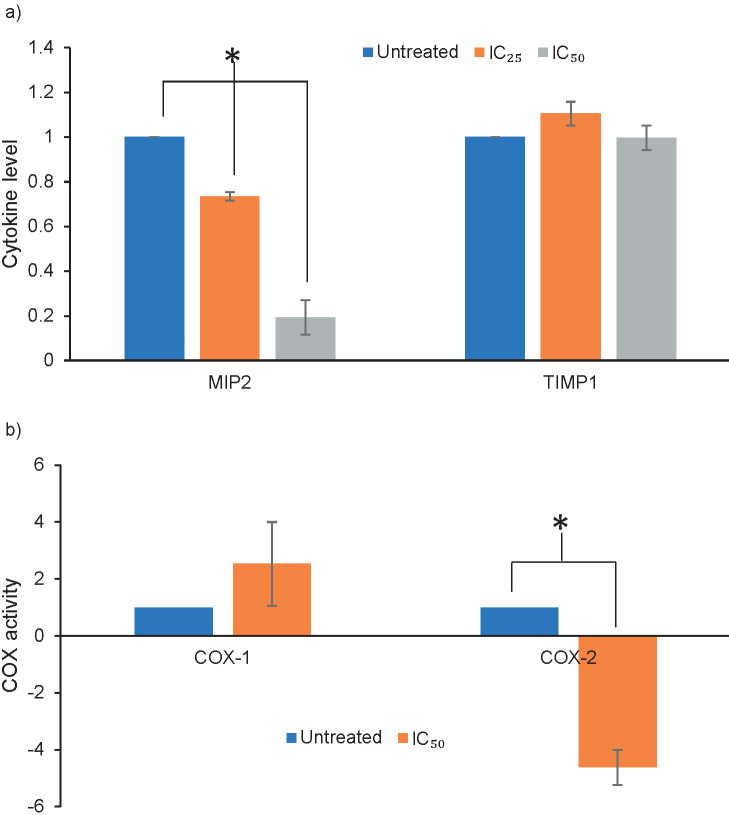
Regulation of modulators in high molecular mass (HMM) extract-treated ORL-204 cells: a) regulation of cytokine (MIP2 and TIMP1) release. Cells were treated with 10 (IC_25_) and 40 µg/mL (IC_50_) of HMM extract for 72 h, and b) effects of HMM extract on cyclooxygenase COX-1 and COX-2 activities in ORL-204 cells. Cells were treated with 40 µg/mL (IC_50_) of HMM extract for 72 h. Values are expressed as mean±S.D. (*N*=3; *p<0.05)

A previous work done using wild type *L. rhinocerus* revealed a novel water-soluble polysaccharide-protein complex that could potentially be immunomodulatory agent for cancer immunotherapy (*34*). Sum *et al.* (*28*) have reported that *L. rhinocerus* TM02® regulated the release of several cytokines/chemokines which are associated with tumourigenesis by RAW 264.7 murine macrophages, in particular the MIP2 and TIMP1. We proceeded to question if TM02® also demonstrated comparable immunomodulating properties in oral cancer in addition to its selective antiproliferative property. We investigated the regulation of the release of these cytokines in ORL-204 over a period of 72 h after treatment with HMM extract at 10 (IC_25_) and 40 µg/mL (IC_50_). HMM extract significantly inhibited the release of MIP2 from ORL-204 by 30 to 80% in a dose-dependent manner, while no effect was observed for TIMP1 (Fig. 4a). In most cancers, MIP2 expression is upregulated for cell proliferation promotion and metastasis (*35*). Its secretion inhibition by HMM extract therefore suggests a repressive effect of the extract towards ORL-204 growth, while its associative role in the alteration of osteoclastic activity remains unknown. It has been reported that TNF-α mediated the increase of MIP2 mRNA *via* NF-κB/MAPK, a caspase-3 signalling pathway in macrophages (*36*), but the question whether similar mechanisms are applicable to our current study remains to be answered. However, in view of its anticancer effect by promoting caspase-3/7 activities and suppressing MIP2 secretion, it is suggestive that HMM extract has antagonizing and/or multiple involvements in the TNF signalling pathway.

Many of the cytokines and mediators of inflammatory pathways are involved in the different steps of tumourigenesis (*37*). Non-steroidal anti-inflammatory drugs (NSAIDs) have been reported to prevent cancer and stop tumour growth by inhibiting prostaglandin synthesis through COX-2 hindrance (*38*). We further investigated the potential role of COX in regulating the inhibitory effect of HMM extracts on ORL-204. Cells were treated with HMM extract at IC_50_ and the COX levels were determined (Fig. 4b). The selective inhibition of HMM extracts towards COX-2 might indicate its potential role against the development of oral lesions by inhibiting and/or interrupting the oral carcinogenesis pathway, further strengthening the therapeutic potential of COX-2 inhibitors in oral cancer treatment (*39*). There has also been increasing evidence that COX-2 produced prostaglandins that intervene in tumour cell proliferation while some selective COX-2 inhibitors such as nabumetone inhibit proliferation of various COX-2-expressing cancer cells by a G_0_/G_1_ phase cell cycle arrest (*40*). Our current findings point to a possible linkage between COX-2 inhibitory effect and G_0_/G_1_ phase arrest in ORL-204. However, due to the limitations of an *in vitro* study, the association remains to be elucidated.

**Fig. 5:**
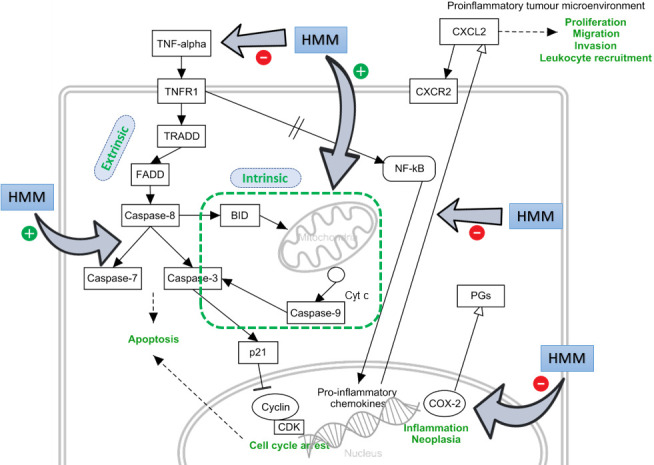
Proposed tumour necrosis factor (TNF) cell signalling pathway intervention by high molecular mass fraction (HMM) in different phases. Anticancer effects of HMM extract in ORL-204 *via* apoptosis, proliferation inhibition (cell cycle arrest), immunomodulation and anti-inflammation (*41*) by means of targeting multiple cell signalling pathways and transcription factors. TNFR1=tumour necrosis factor receptor 1, TRADD=TNFR1-associated death domain, FADD=Fas associated with death domain, BID=BH3 interacting-domain death agonist, Cyt c=cytochrome complex, PGs=prostaglandins, CXCL2=chemokine (C-X-C motif) ligand 2 (MIP2 equivalent), CXCR2=CXC chemokine receptor 2

From this study, we showed that HMM extract of *L. rhinocerus* induced apoptosis in ORL-204 cells *via* the activation of caspase-3/7 through the extrinsic and intrinsic signalling pathways. HMM extract further manipulated cell cycle by arresting the cells at G_0_/G_1_ phase. Several molecules such as MIP2 and COX-2 related to TNF signalling contributed to the anticancer effect of HMM extract towards ORL-204, where both immunomodulators have been implicated in cell proliferation and inflammation (Fig. 5). A halted COX-2 expression has been shown to decrease MIP2 (*41*). Lee *et al.* (*42*) also reported that HMM extract attenuated TNF-α activity in lipopolysaccharide-induced RAW 264.7 cells, signifying an anti-inflammatory/immunosuppressive effect. HMM extract contains abundant amounts on dry mass basis of carbohydrates and proteins (4%) (*42*), suggesting the existence of polysaccharide-protein complexes in the fraction. The conglomeration of molecules in HMM extract may have contributed to its diverse roles in targeting various signalling pathways such as apoptosis, inflammation and immunomodulation in order to exert its anticancer effect.

## CONCLUSIONS

This is the first study to reveal the capability of *Lignosus rhinocerus* TM02® in aiding oral cancer treatment and/or as a form of preventive measure against tumourigenesis by the intervention of the tumour necrosis factor (TNF) signalling. The action of biting/chewing the sclerotium, which has been practiced traditionally, may now have an insightful implication. Cold water extract of the tiger milk mushroom demonstrated selective cytotoxic effect to ORL-48 and ORL-204 cell lines, with little cytotoxicity towards primary human fibroblast, while high-molecular mass polysaccharide-protein complexes from the cold water extract induced apoptosis and exhibited antiproliferative activity against ORL-204 by G_0_/G_1_ phase cell cycle arrest and inhibition of several immunomodulators affiliated with the TNF signalling pathway, such as MIP2 and COX-2. Further investigations including *in vivo* and downstream molecular works are warranted to strengthen the current findings. As a limitation of this study, future work will also incorporate anticancer drug(s) as positive control to test along with purified fractions.
